# Evaluation of anxiety and coping strategies among tunisian health professionals in the pandemic of the covid 19

**DOI:** 10.1192/j.eurpsy.2021.790

**Published:** 2021-08-13

**Authors:** N. Messedi, A. Chamseddine, O. Bouattour, M. Turki, N. Halouani, J. Aloulou

**Affiliations:** Psychiatry, Hedi Chaker University hospital, sfax, Tunisia

**Keywords:** covid 19, caregivers, anxiety, coping, covid 19, caregivers, Anxiety

## Abstract

**Introduction:**

The rapid spread of coronavirus has forced the healthcare systems in Tunisia to reorganize its structures, thus mobilizing all caregivers. Their professional and emotional burden was put to the test.

**Objectives:**

To evaluate the level of anxiety and to study coping strategies among caregivers during this pandemic.

**Methods:**

A cross-sectional descriptive and analytical study among150 caregivers in two hospitals in Sfax in Tunisia; during April 2020. We used anonymous questionnaire, the Spielberger State Anxiety Scale(STAI) to assess tension felt at anxiety-producing situations; and the Coping Inventory Scale for Stressful Situations (CISS): to assess coping strategies.

**Results:**

The average age was 30.33± 6,93 years and the sex-ratio M/W = 0,29. Caregivers followed the news of this pandemic with these means of communication: 96% Facebook, 80%TV. The increase of the time spent in front of media:84% Sleep disorders were present in 64.7%: insomnia (36%), chopped sleep(34%). Caregivers used sleeping pills in 12% of case. STAI: The mean ascore =48.85 and a high anxiety level was noted in72% of case. CISS: Task-oriented coping strategies : a mean score= 47.90 and Emotion-centered coping : a mean score = 40,49 High anxiety was correlated with: age>40 years old (p=0.042). The increase of the time spent in front of media, chopped sleep and use of sleeping pills are correlated respectively (p= 0,043, p=0,003, p=0,003) with an emotionally focused coping strategy.

**Conclusions:**

Health professionals had a painful psychological experience with significant anxiety. Strengthening prevention strategies, management of health crises should be a priority of our health-system.

